# Experimental bed rest as a model to investigate mechanisms of, and countermeasures against, microgravity and disease‐free inactivity

**DOI:** 10.1113/EP091795

**Published:** 2024-03-13

**Authors:** Rodrigo Fernandez‐Gonzalo, Colleen S. Deane, Damian Miles Bailey

**Affiliations:** ^1^ Department of Laboratory Medicine, Division of Clinical Physiology Karolinska Institutet Stockholm Sweden; ^2^ Unit of Clinical Physiology Karolinska University Hospital Stockholm Sweden; ^3^ Human Development & Health, Faculty of Medicine University of Southampton, Southampton General Hospital Southampton UK; ^4^ Neurovascular Research Laboratory, Faculty of Life Sciences and Education University of South Wales Pontypridd UK

**Keywords:** bed rest, inactivity, microgravity, spaceflight

1

More humans are now entering space than ever before, owing to significant investment from governmental and commercial agencies looking ambitiously to expand the reach of humanity beyond low Earth orbit, involving habitation of a permanent base on the surface of the Moon ahead of the horizon goal, a crewed mission to the red planet, Mars. With the advent of long‐duration interstellar travel, there is mounting pressure to gain a better understanding of the functionally integrated physiological responses to, and countermeasures that mitigate against, the maladaptive changes incurred by microgravity and physical inactivity. Owing to the technical, logistical and financial costs associated with conducting spaceflight research, 6° head‐down‐tilt bed rest (HDBR), first introduced by a Soviet team led by Genin and Kakurin (1972), has become the mainstay ground‐based analogue of microgravity (Hargens & Vico, 2016).

In this issue of *Experimental Physiology*, Hajj‐Boutros et al. (2024) comprehensively detail the first Canadian study (supported by the Canadian Space Agency, Canadian Institutes of Health Research and Canadian Frailty Network) that seeks to characterize the physiological, psychological and cognitive responses to 14 days of HDBR and to assess the adaptive benefits of exercise during HDBR. This national effort, composed of eight research teams, involved the recruitment of 23 older males and females (55–65 years), who completed 14 days of HDBR, followed by 7 days of recovery (supervised rehabilitation in all participants). During HDBR, half of the participants performed aerobic (high‐intensity interval training or low‐intensity cycling) and resistance exercise (upper and lower body, 3 sets of 12–15 repetitions at a personalized intensity) in the HDBR or horizontal position three times daily for 60–75 min in total, to test its efficacy as a countermeasure. Participants completed a battery of tests conducted at baseline, during HDBR, during the recovery period and at 4 weeks and 4 months post recovery, involving collection of demographics, cardiorespiratory fitness, bone health, body composition, quality of life, mental health, cognition, muscle health and biological samples for subsequent metabolic and molecular analysis (e.g., mitochondrial function, immunohistochemistry, RNA and protein analysis) (Hajj‐Boutros et al., 2024).

Although many previous studies have been conducted using HDBR (highlighted by e.g., Hargens & Vico, 2016; Kehler et al., 2019), this study stands out owing to the comprehensive nature of the many multidisciplinary measures taken at numerous time points, providing important insight into the temporal kinetics underlying the integrative physiological responses across the functional continuum of adaptation to maladaptation. This study extends the authors’ prior findings focused on HDBR‐induced changes in body composition (Hajj‐Boutros et al., 2023), muscle strength (Hajj‐Boutros et al., 2023), baroreflex responses (Sadeghian et al., 2022) and cardiovascular function (Hajj‐Boutros et al., 2023) and will continue to inform the molecular mechanisms of HDBR and efficacy of exercise as a potential countermeasure. Their findings might also help to standardize/optimize sample collection methodologies that can be adopted by future researchers to reduce biological heterogeneity and create ‘big data’ repositories that can better inform future countermeasure discovery and development.

Hajj‐Boutros et al. (2024) emphasize the relevance of the bed rest model to identify physiological events triggered by microgravity or severe inactivity and the underlying mechanisms driving these changes. Since the pioneering study by Deitrick, Whedon, and Shorr some three‐quarters of a century ago, in which medical students were placed in (horizontal) bed for 6–7 weeks (Deitrick et al., 1948), the bed rest model has proved its worth as an excellent platform to test systemic and local responses to disease‐free inactivity or microgravity. Indeed, well‐conducted bed rest studies make it possible to control the majority of external variables that would otherwise be considered potential confounders, including temperature, light–dark cycles, humidity, oxygen/carbon dioxide partial pressures, diet, activity and sleep. This level of experimental control including detailed longitudinal sampling is unique, notwithstanding obvious limitations given that it is impossible for any ground‐based analogue to replicate deep space flight ‘fully’.

Experimental bed rest is not only a ground‐based analogue of microgravity but is also a highly relevant clinical model reflecting periods of bed rest that occur alongside surgery, injury or illness. As such, deciphering the mechanisms governing adaptation to HDBR might provide unique insight into the processes underlying deconditioning, which is especially relevant in older adults, who are more susceptible to periods of hospitalization (Kehler et al., 2019). Moreover, interventions shown to be effective in the HDBR model could be expected to translate into the clinic, improving the physiological and psychosocial outcomes of patients and older adults.

Another very important and positive feature of the bed rest model is that it has proved to be a useful framework to test countermeasures that mitigate against microgravity and/or severe inactivity‐induced maladaptation. Indeed, exercise, nutritional, psychological and pharmaceutical countermeasures can be implemented with a level of control unparalleled by any other human research model. Clinical trials with a control group (bed rest only) and an intervention group (countermeasure) provide unique details about the efficacy and feasibility of the countermeasure being tested. However, given the inherent logistics and cost implications, the ability to compare multiple countermeasures within the same study is understandably limited. Indeed, only the major space agencies can prepare, fund and perform these types of specialist experiments. For example, the European Space Agency is currently conducting two 60‐day bed rest studies (i.e., BRACE: Bed Rest with Artificial gravity and Cycling Exercise, and BRAVE: Bed Rest with Artificial gravity and Vibration and resistance Exercise), each with three study arms: bed rest only, bed rest and exercise, and bed rest and exercise and artificial gravity. Only with this design, or with the advent of global standardization as highlighted before, can future researchers identify which countermeasure is potentially ‘optimal’ to safeguard crew health and performance, an obligatory requirement for spaceflight beyond low Earth orbit.

In conclusion, the study by Hajj‐Boutros et al. (2024) demonstrates that it is feasible to conduct a comprehensive longitudinal bed rest study offering unique temporal and mechanistic insight in older participants. This integrative approach will allow the research teams to investigate the effects of bed rest and the countermeasures adopted in most of the physiological systems of the human body. It is also important to highlight that a great part of the successful implementation of this Canadian endeavour is the involvement of multiple collaborators with different backgrounds, creating a dynamic and efficient multidisciplinary environment, with the whole being more than the sum of its parts!

## AUTHOR CONTRIBUTIONS

All authors have read and approved the final version of this manuscript and agree to be accountable for all aspects of the work in ensuring that questions related to the accuracy or integrity of any part of the work are appropriately investigated and resolved. All persons designated as authors qualify for authorship, and all those who qualify for authorship are listed.

## CONFLICT OF INTEREST

Damian Bailey is Editor‐in‐Chief of Experimental Physiology, Chair of the Life Sciences Working Group, member of the Human Spaceflight and Exploration Science Advisory Committee to the European Space Agency, member of the Space Exploration Advisory Committee to the UK Space Agency, member of the National Cardiovascular Network for Wales and South East Wales Vascular Network and is affiliated to the companies FloTBI, Inc. and Bexorg, Inc. focused on the technological development of novel biomarkers of cerebral bioenergetic function and structural damage in humans.
